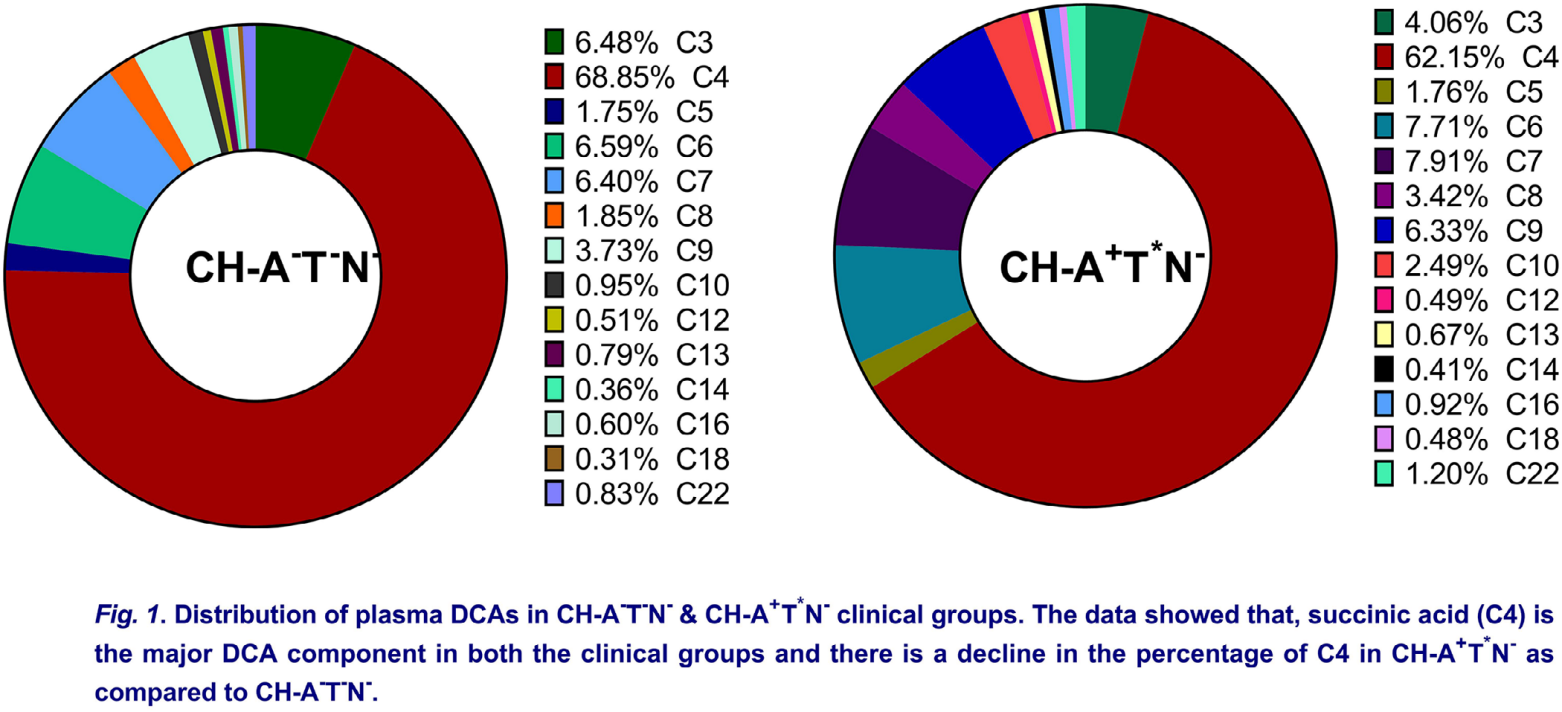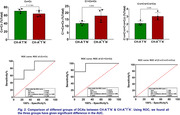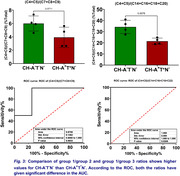# Plasma dicarboxylic acids as indicators of impaired energy metabolism and lipid oxidation associated with pre‐symptomatic Alzheimer’s disease

**DOI:** 10.1002/alz.091933

**Published:** 2025-01-09

**Authors:** Joby Jose, Alfred N. Fonteh

**Affiliations:** ^1^ Huntington Medical Research Institutes, Pasadena, CA USA

## Abstract

**Background:**

Dicarboxylic acids (DCAs) are critically important for intermediate metabolism. Since mitochondrial dysfunction and energy dysregulation are associated with AD pathology, we hypothesize that fluctuations in plasma DCAs would accompany AD pathology.

**Method:**

In an ongoing brain‐aging study, we recruited older adults (>65 years) classified as cognitively healthy (CH) after neuropsychological testing. Based on the CSF biomarker analysis, we further categorized them as CH participants with normal biomarker levels (CH‐A^‐^T^‐^N^‐^) and CH participants with AD‐like pathological biomarker levels (CH‐A^+^T^*^N^‐^) having a high risk of cognitive decline (presymptomatic AD). We collected plasma from the participants and extracted DCAs using ethyl acetate. We derivatized the DCAs using dimethylaminophenacyl bromide (DmPABr) to reverse the DCA polarity and enhance the sensitivity of detection. DCA levels were quantified using isotope dilution liquid chromatography mass spectrometry with multiple reaction monitoring of C3‐C22 DCAs. The group differences between CH‐A^‐^T^‐^N^‐^ and CH‐A^+^T^*^N^‐^ were calculated using Mann‐Whitney comparison tests.

**Result:**

Distribution studies revealed that most of the dicarboxylic acids in the plasma samples (n = 4) are short‐chain (≈80%) and medium‐chain DCAs (≈15%), while long‐chain DCAs make less than 5% (Figure 1). We compared the distribution of plasma DCAs in CH‐A^‐^T^‐^N^‐^ versus CH^‐^A^+^T^*^N^‐^ and identified three groups. The DCA composition of group 1 DCAs (C4+C5) was lower in CH‐A^+^T^*^N^‐^ than in CH‐A^‐^T^‐^N^‐^. In contrast, the DCA levels of group 2 (C7+C8+C9) and group 3 (C14+C16+C18+C22) were higher in the CH‐A^+^T^*^N^‐^ plasma compared to CH‐A^‐^T^‐^N^‐^. Using ROC, significant differences in the AUC were shown for each of the three groups (Figure 2). The group differences resulted in significantly higher group 1/group 2 and group 1/group 3 ratios in CH‐A^‐^T^‐^N^‐^ compared to CH‐A^+^T^*^N^‐^ (Figure 3).

**Conclusion:**

We identified 14 DCA species in plasma and showed that their plasma composition varied with AD biomarker levels. DCAs associated with energy (group 1) were higher in CH‐A^‐^T^‐^N^‐^ than in CH‐A^+^T^*^N^‐^, while DCAs linked with longer‐chain fatty acid oxidation (group2 & 3) were higher in CH‐A^+^T^*^N^‐^ than in CH‐A^‐^T^‐^N^‐^. These data suggest that there is dysregulation of DCA metabolism in pre‐symptomatic AD, and plasma levels of DCAs can be used as biomarkers of early‐stage AD.